# A Randomized Controlled Neurophysiological Study of a Chinese *Chan*-Based Mind-Body Intervention in Patients with Major Depressive Disorder

**DOI:** 10.1155/2013/812096

**Published:** 2013-12-30

**Authors:** Agnes S. Chan, Yvonne M. Y. Han, Sophia L. Sze, Queenie Y. Wong, Mei-chun Cheung

**Affiliations:** ^1^Department of Psychology, The Chinese University of Hong Kong, Shatin, Hong Kong; ^2^Chanwuyi Research Center for Neuropsychological Well-Being, The Chinese University of Hong Kong, Shatin, Hong Kong; ^3^Henan Songshan Research Institute for Chanwuyi, Henan 452470, China; ^4^Department of Special Education and Counselling, The Hong Kong Institute of Education, Tai Po, Hong Kong; ^5^Division II, Kwai Chung Hospital, Kwai Chung, Hong Kong; ^6^Department of Social Work, The Chinese University of Hong Kong, Shatin, Hong Kong

## Abstract

Our previous studies have reported the therapeutic effects of 10-session Chinese *Chan*-based *Dejian* mind-body interventions (DMBI) in reducing the intake of antidepressants, improving depressive symptoms, and enhancing the attentional abilities of patients with depression. This study aims to explore the possible neuroelectrophysiological mechanisms underlying the previously reported treatment effects of DMBI in comparison with those of cognitive behavioral therapy (CBT). Seventy-five age-, gender-, and education-matched participants with depression were randomly assigned to receive either CBT or DMBI or placed on a waitlist. Eyes-closed resting EEG data were obtained individually before and after 10 weeks. After intervention, the DMBI group demonstrated significantly enhanced frontal alpha asymmetry (an index of positive mood) and intra- and interhemispheric theta coherence in frontoposterior and posterior brain regions (an index of attention). In contrast, neither the CBT nor the waitlist group showed significant changes in EEG activity patterns. Furthermore, the asymmetry and coherence indices of the DMBI group were correlated with self-reported depression severity levels and performance on an attention test, respectively. The present findings provide support for the effects of a Chinese *Chan*-based mind-body intervention in fostering human brain states that can facilitate positive mood and an attentive mind.

## 1. Introduction

Mind-body interventions focus on the interaction between the brain, the mind, and the body with the assumptions that the mind and body are interconnected and that individuals can use the mind to affect physical functioning and mental health. The use of mind-body training has received increasing interest as a complementary intervention method among psychologists and medical professionals due to its documented therapeutic effects on many psychological problems, such as anxiety [1], insomnia [2], and depression [3]. Mind-body training has also been shown to have positive effects as a complementary treatment for many physical disorders, including irritable bowel syndrome [4], chronic pain [5], and cardiovascular problems [6]. Using the mind to affect physical and mental health is a core concept of traditional Chinese medicine. Studies assessing the effect of *qigong*, a mind-body practice from ancient China, have demonstrated that *qigong* can elevate mood in elderly people with depression [7] and improve the psychological wellbeing and self-efficacy of patients with chronic physical illnesses [8]. *Tai Chi-*, one of the best known and widely studied mind-body exercises, originated in China and has been found to be effective in reducing anger, mood disturbances, somatic symptoms, and sleep problems in patients with chronic conditions [9, 10].

Recently, a Chinese *Chan*-based mind-body intervention, termed “*Dejian* Mind-Body Intervention” (DMBI), has begun to be applied in clinical and educational settings as a complementary treatment for different psychological and cognitive disorders. This intervention is based on a medical principle that originated from the *Shaolin *Temple, is holistic in nature, and consists of three integrative treatment components: (i) psychological education, (ii) mind-body exercise, and (iii) diet modification [11, 12]. Empirical evidence has shown that the DMBI has beneficial effects on mood and cognitive problems related to brain disorders. Specifically, a randomized controlled study showed positive effects of one month of DMBI training on depressive mood in a group of community-dwelling adults [13]. Clinical studies of patients with developmental or acquired brain disorders who practice DMBI have also reported significant improvements in measures of cognitive function and substantial reductions of problem behaviors [14–19].

**Figure 1:**
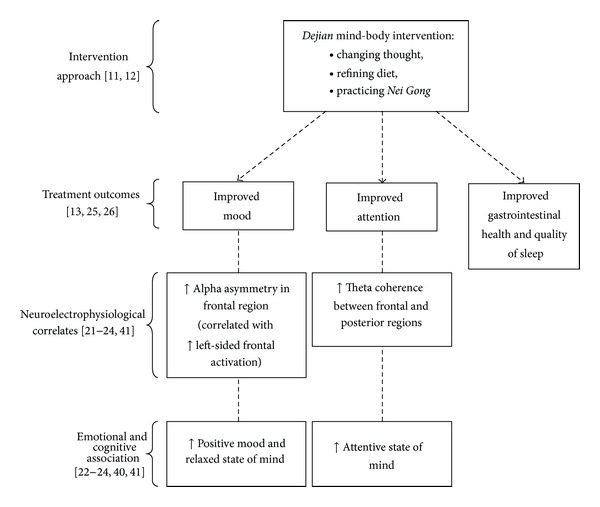
Summary of empirical evidence for the potential neural mechanism underlying the effects of *Dejian* mind-body intervention (DMBI). Dotted arrows represent empirically found treatment effects of DMBI, whereas dotted lines indicate hypothetical linkage between possible neuroelectrophysiological correlates and changes in mood and cognitive states.

Some researchers attribute the reported therapeutic effects of mind-body interventions to the acute effects of complex mental processes that involve changes within the central nervous system (CNS) [20]. Indeed, the advent of brain imaging techniques has helped to address the fundamental question about the relationship between the mind and the brain. A recent electrophysiological study, that measured the electroneural activities associated with the DMBI mind-body exercise, which consists of the passive and active subtypes of *Dan Tian *breathing techniques [21], found that passive *Dan Tian* breathing induces a relaxed and calm state of mind as indicated by elevated EEG alpha asymmetry and that active *Dan Tian* breathing induces an attentive state of mind as indicated by elevated EEG theta coherence. These results are consistent with previous electrophysiological studies of affective processes that have shown that positive emotion is coupled with greater left-sided anterior activation as indexed by decreased alpha activity in the left hemisphere [22] and that enhanced attention and working memory are associated with elevated EEG theta coherence [23, 24]. The potential effects of DMBI on the CNS have been summarized in Figure 1.

The findings that the mind-body exercise of DMBI is able to elicit a specific state of consciousness in which relaxation and internalized attention coexist may provide an explanation of its therapeutic effects of reducing stress and improving cognitive function in patients with depression, group known to have attention problem and be more stress-prone; this brain state enables individuals to remain focused on a task, be free of distractions, and deal with life stressors in a relaxed and flexible manner. This idea is supported by one of our recent studies of patients with clinical depression ([25]; see Figure 1); in this study, 75 participants diagnosed with major depressive disorder were randomly assigned to receive 10 sessions of cognitive behavioral therapy (CBT) or 10 sessions of DMBI or placed on a waitlist. Pre-post measurements included the use of antidepressants, ratings from psychiatrists who were blinded to the experimental design, self-reported mood measures, and performance on an attentional task. The results showed that, although both the CBT and DMBI groups demonstrated significantly reduced overall depressive symptoms after the intervention with large effect size (0.93–1.10), only the DMBI group demonstrated a significant reduction in the intake of antidepressants and improvements in some common depression-related cognitive and physical symptoms, such as attention deficits and gastrointestinal problems. In addition to the reduction of depressive moods with DMBI, a related study of patients with depression practicing DMBI also reported substantial improvement in the patients' sleep patterns as measured by significant reductions in psychiatrists' ratings of overall sleep problems and total sleep time relative to waitlist or CBT controls ([26]; see Figure 1).

In sum, the results of our previous studies support the idea that DMBI is effective in improving mental and physical functioning. However, the neurophysiology of this mind-body intervention remains elusive. Thus, the purpose of the present study was to investigate how DMBI affects the function of the CNS of patients with depression, with a specific focus on the mental processes of positive emotion and attention. Given that positive emotion has been reported to be related to greater left-side anterior activation, as indexed by decreased EEG alpha activity in the left hemisphere, and attention has been linked to greater EEG theta coherence, we hypothesized that this *Chan*-based mind-body intervention would result in EEG changes involving increased left-sided anterior activation and increased theta coherence compared to controls. We also hypothesized that increased left-sided anterior activation would be associated with improved depressive symptoms and that increased theta coherence would be associated with improved attention in the patients practicing DMBI.

To further highlight DMBI's therapeutic effect of inducing a specific state of consciousness in which both relaxation and internalized attention coexist, we compared patients who received DMBI to CBT and waitlist controls. CBT has been empirically demonstrated to reduce depressive moods in people with depression by helping depressed individuals to reinterpret their environment and their interactions with others in a positive and realistic manner [27, 28]. We anticipated that both DMBI and CBT would induce increased left-sided anterior activation suggestive of reductions in depressive moods compared to the waitlist controls. However, we expected that only the DMBI would induce increased theta coherence, which is suggestive of focused internalized attention.

## 2. Materials and Methods

### 2.1. Participants

Seventy-five participants were recruited from the outpatient clinic at the West Kowloon Psychiatric Centre. All participants had received diagnoses of major depressive disorder from a clinical psychologist based on the diagnostic criteria of the Diagnostic and Statistical Manual of Mental Disorders (DSM-IV-TR) [29] and the Structural Clinical Interview for the DSM-IV (Chinese-bilingual SCID-I/P version) [30]. Exclusion criteria included positive history of head injury, seizure, stroke, other CNS diseases, other comorbid psychiatric illness, or reports of strong suicidal ideation. All participants were prescribed antidepressants and were continuously followed up by their psychiatrists, who were not coinvestigators and were blind to the group assignment and rationale of the present study. Randomization of the assignment of participants into the CBT, DMBI, or waitlist control groups was conducted by a medical professional who was blind to the experimental design. Participants in the two intervention groups were blind to the potential benefits of the two techniques.

Participants who dropped out of the study or attended less than 70% of the intervention sessions were excluded from analyses. After intervention, 16, 17, and 17 participants remained in the waitlist control, CBT, and DMBI groups, respectively. The average attrition rate (inclusive of dropouts and participants with <70% attendance rate) of the three groups is 33%, which is comparable to the average range of attrition rate of psychotherapy for patients with depression [31]. Reasons for the dropouts and low attendance rate included unexpected change in work schedule or other personal commitments that have clashed with the training or assessment sessions.  Table 1 presents the demographic characteristics of each group of participants at baseline. The three groups were matched for age, *F*(2, 47) = 0.198, *P* = 0.821; education level, *F*(2, 47) = 1.729, *P* = 0.189; and gender, *χ* ^2^(2) = 1.103, *P* = 0.576. Across groups, depression severity, as reflected by the SCID-based diagnoses, *χ* ^2^(4) = 3.133, *P* = 0.536; illness duration, *F*(2, 46) = 0.426, *P* = 0.656; and total scores on the Chinese version of the Beck Depression Inventory (BDI-II) [32, 33], *F*(2, 47) = 0.155, *P* = 0.857, was similar.

### 2.2. Ethics

The study was conducted in accordance with the Helsinki Declaration of the World Medical Association Assembly. The research protocol was approved by the Joint Chinese University of Hong Kong-New Territories East Cluster (Joint CUHK-NTEC) Clinical Research Ethics Committee (CREC) and the Kowloon West Cluster (KWC) CREC of the Hospital Authority in Hong Kong SAR.

### 2.3. Procedure

Written informed consent was obtained from each participant prior to the study. Each participant's state of depression was individually assessed by research assistants using a standardized questionnaire, attention abilities, and EEG activities at baseline and again after 10 weeks. The depression severity was measured with the 21-item BDI-II, which the participants themselves completed. The total maximum score of the BDI-II is 63, where higher score indicates more severe depressive symptoms. The total BDI-II scores can be classified into four severity levels: “Normal (0–13 points),” “Mild Depression (14–19 points),” “Moderate Depression (20–28 points),” and “Severe Depression (29–63 points).” Attention abilities were assessed with the Digit Vigilance Test (DVT) [34], which requires participants to search for target digit “6” or “9” within an array of other distracting digits as quickly as possible. The total time taken to complete the task is the outcome measure, and shorter completion times suggest greater work efficiency and increased ability to maintain attention on the task. The EEG data were collected from each participant using the TruScan measuring set with 19 electrodes positioned across the scalp according to the International 10–20 System [35] in a sound- and light-attenuated room before and after intervention. During each measurement time point, the participants were required to follow through a brief video introducing the* Dan Tian* Breathing technique, after which they were required to perform the *Dan Tian* Breathing for five minutes. Then, they were asked to sit with their eyes closed for another five minutes, during which their resting state EEG data were captured for subsequent analyses. It should be noted that participants from all three groups have undergone the same experimental paradigm, and only participants from the DMBI group had continuous daily practice of the *Dan Tian* Breathing during the intervention period. The pre-post comparison on the EEG changes for each group will provide clue to the potential effects of regular practice of *Dan Tian* Breathing in DMBI and to minimize potential placebo effect. All electrode impedances were kept at 10 kΩ or below. The EEG signals were referenced to linked ears, digitally filtered at 0.5 and 100 Hz, and sampled at 256 samples per second with a high-frequency band pass limit of 30 Hz. Artifacts were removed off-line based on records of the bodily movements of the participants made by the experimenter throughout the EEG assessments. After the baseline assessment, the two treatment groups underwent 10 weekly 90-minute training sessions of either CBT or DMBI, and the waitlist control group did not receive any psychological intervention.

### 2.4. Intervention

The two intervention groups were designed to have similar structures and formats with respect of group size, the venue of intervention, the duration and frequency of the sessions, didactic teaching on the fundamental principles and corresponding techniques, in-session sharing, and home assignments. The CBT group was run by a clinical psychologist with over ten years of clinical experience conducting this type of group training regularly in a hospital. Another clinical psychologist who also has over ten years of clinical experience and developed DMBI ran the DMBI group. Participants in each intervention group were closely monitored in terms of their abilities to master the techniques by the respective therapists throughout the intervention period.

#### 2.4.1. DMBI

This intervention was developed based on a Chinese* Chan *tradition called *Chanwuyi* (i.e., Zen, martial arts, and healing) that originated from the *Shaolin* Temple. This intervention was named after the Grand Master of *Chanwuyi*—*Shi Dejian* (a *Shaolin* monk). The principle of DMBI is to alleviate psychological distress by understanding the root of problems in accordance with Buddhism philosophy, enhance mental and physical health by practicing some of the *Shaolin* mind-body exercises (called *Nei Gong*), and uphold the concept of “food as medicine.” The DMBI proposed to refine the diet to reduce the intake of high-fat and high-energy food that will generate excessive heat inside the body and cause blood and *Qi* stagnation, which in turn would have harmful effects on physical and mental health [17]. DMBI emphasizes integrative treatment for the mind and the body, which, on the one hand, changes the thought process and, on the other hand, improves mental and physical health via the practice of mind-body exercises and refining of the diet.

Details of the intervention regime have been elaborated in our previous studies [25, 26]. Briefly, the treatment has four components: (1) increase awareness of how greed, anger, and obsession affect our mental and physical health to guide changes that reduce psychosomatic problems; (2) consume seven categories of food (fresh vegetables, fruits, grain, beans, mushrooms, nuts, and root vegetables) that are healthy and reduce intake of some foods that generate internal heat (ginger, garlic, green onion, spicy foods, eggs, meat, and fish); (3) practice self-awareness and self-control; and (4) practice *Nei Gong *(the mind-body exercises) to reduce stress, increase flexibility in the four limbs, enhance strength of the legs, improve overall physical health, and improve the circulation of *Qi* and blood. A key of DMBI is that individuals are encouraged to execute their treatment regime naturally in adherence with their own lifestyles and plans and to feel the changes their minds and bodies undergo in response to changes in their thoughts, diet, and exercise habits. For instance, participants were not required to instantly abstain from all hot and spicy foods; rather, they were encouraged to gradually reduce their intake of these foods. Furthermore, there were no constraints regarding the time spent practicing *Nei Gong.* Participants were instructed to practice the exercises until they felt warm and relaxed and not to overexert themselves. The rationale behind this flexibility in the practice of DMBI was to facilitate increases in the participants' senses of self-control and self-awareness.

#### 2.4.2. CBT

The present study adopted a CBT protocol that was developed based on three sources: *Cognitive Therapy for Depression *[36], *Mind Over Mood* [37], and *Cognitive Behavioural Therapy in Groups* [38]. This protocol covers the typical components of CBT, including progressive muscle relaxation (PMR), behavioral activation, self-monitoring, cognitive restructuring, rehearsal of coping skills, and relapse prevention. The intervention began with psychoeducation on the biopsychosocial model of depression and the triadic relationship between mood state, cognition, and behavior. Through the introduction of the triadic relationship, the participants were guided toward positively modifying their cognitive processes and behavior so as to trigger a positive change in mood. Furthermore, the participants were taught to practice PMR as a means to induce a relaxed state of mind. PMR is a stress management technique developed by Jacobson [39] and is a widely practiced relaxation method that has been reported to be effective in alleviating anxiety and mood problems [40]. Participants were taught to alternately tense and relax groups of muscles in a prescribed sequence from the head to the feet according to the standard procedure of the technique. Participants were also instructed to synchronize their breathing with the muscle-tensing/relaxing cycle and concurrently feel their bodily changes. Throughout the sessions, the therapist reviewed the experiences of participants during their daily practice of the techniques and closely monitored their progress.

### 2.5. EEG Indices

#### 2.5.1. Frontal Alpha Asymmetry

The absolute power in the alpha frequency band (8–13 Hz) measured from the anterior scalp region (F7 and F8) was natural logarithm (ln)-transformed and used to calculate frontal alpha asymmetry. The asymmetry index was computed as (ln (F8)−ln (F7)), where F8 and F7 are the alpha powers in the right and left hemispheres, respectively. EEG alpha power has been reported to be negatively correlated with brain activation [41, 42]. Therefore, positive frontal alpha asymmetry values resulting from greater alpha power on the right and less alpha power on the left would represent greater activity in the left relative to the right brain (i.e., left-sided activation), whereas negative asymmetry values would suggest greater right side activation. The alpha asymmetry in the anterior frontal (F7 and F8) region was used given its reported association to emotions, of which greater left-sided activation was observed to be coupled with positive mood and relaxed mind [22].

#### 2.5.2. Intra- and Interhemispheric Theta Coherence

Coherence values in the theta band (3–7 Hz) measured at the intra- and interhemispheric connections within and between the frontal (F3, F4, F7, and F8) and posterior (P3, P4, O1, and O2) scalp regions were obtained, and the square rooted values were normalized using Fisher's *Z* transformation. The mean values of six clusters of coherence pairs were computed. These means included intrahemispheric coherence in (i) frontal (F3-F7 and F4-F8), (ii) posterior (P3-O1 and P4-O2), and (iii) frontoposterior regions (F3-P3, F4-P4, F7-P3, F8-P4, F3-O1, F4-O2, F7-O1, and F8-O2) and interhemispheric coherence in (iv) frontal (F3-F4, F7-F8, F3-F8, and F4-F7), (v) posterior (P3-O2, P4-O1, P3-P4, and O1-O2), and (vi) frontoposterior regions (F3-P4, F4-P3, F7-P4, F8-P3, F3-O2, F4-O1, F7-O2, and F8-O1). The specific electrode sites were chosen and grouped based upon previous studies that reported that short- and long-range frontal and posterior theta coherence indices are sensitive to attentional processes [24, 43].

### 2.6. Data Analysis

All data were analyzed with SPSS (version 15.0) statistical software. Baseline group differences in demographic and clinical characteristics were explored with ANOVA and *χ* ^2^ tests. The main analyses of the effects of intervention on various EEG indices were performed using paired-sample one-tailed *t* statistics with planned comparisons. As specific hypotheses were tested, and the number of comparisons was large within a relatively small group of participants, Student's *t*-test was adopted without adjustments to the alpha level so as to avoid lowering the power of the tests. At the same time, effect sizes (Cohen's *d* statistic) were calculated to evaluate the degree of changes from baseline to after intervention. Any between-group differences were analyzed using ANOVA or independent-sample Student's *t* tests. Pearson's correlations and *χ* ^2^ tests were employed to explore possible associations between the variation in EEG indices and depression severity and attentional abilities.

## 3. Results

### 3.1. DMBI Enhanced Frontal Alpha Asymmetry

**Figure 2:**
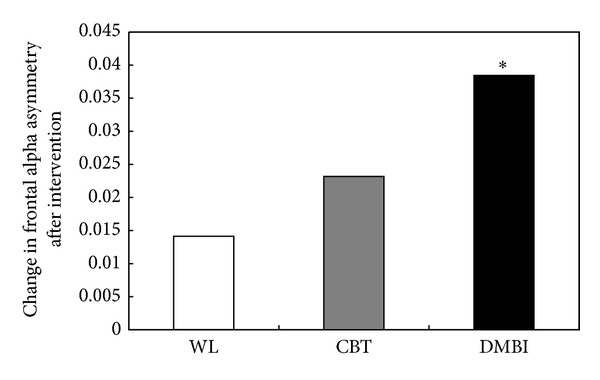
Comparison of the frontal alpha asymmetry after 10-week training between the waitlist, CBT, and DMBI groups. Each bar represents the difference in alpha asymmetry between baseline and postintervention values. Positive values indicate increased level of alpha asymmetry after intervention; negative values indicate a reduction in asymmetry. WL = waitlist group; CBT = cognitive behavioral therapy group; DMBI = *Dejian* mind-body intervention group. *P < 0.05 (paired samples Student's *t*-test).

The ANOVA results revealed that the baseline levels of frontal alpha asymmetry were comparable across the three groups, *F*(2, 46) = 0.894, *P* = 0.416.  Figure 2 shows the changes in alpha asymmetry in each group after intervention. Participants who received DMBI demonstrated significantly enhanced frontal alpha asymmetry after intervention, *t*(15) = 1.866, *P* = 0.041, and effect size = 0.466. Although participants who received CBT showed a trend toward enhancement, and their postintervention alpha asymmetry levels were intermediate to those of the waitlist control and DMBI groups, enhancement in this group did not reach statistical significance, *t*(16) = 1.147, *P* = 0.134, and effect size = 0.279. Participants in the waitlist group showed no significant changes in alpha asymmetry after 10 weeks, *t*(15) = 0.422, *P* = 0.340, and effect size = 0.106. The mean increment of asymmetry level in the DMBI group was 0.038 units (SD = 0.082), which was nearly double that of the CBT group (mean increment = 0.023 units; SD = 0.083) and nearly triple that of the waitlist group (mean increment = 0.014 units; SD = 0.134). Multiple sources of scientific evidence have suggested that greater frontal alpha asymmetry indicates positive mood and a state of relaxation [22, 23, 44, 45]. The significant elevation of frontal alpha asymmetry in the DMBI group implies that DMBI not only improves the mood states of patients with depression, as we have previously reported, but also fosters a more relaxed and “happier” brain state that is accompanied by left frontal activation.

### 3.2. DMBI Enhanced Inter- and Intrahemispheric Theta Coherence

**Figure 3:**
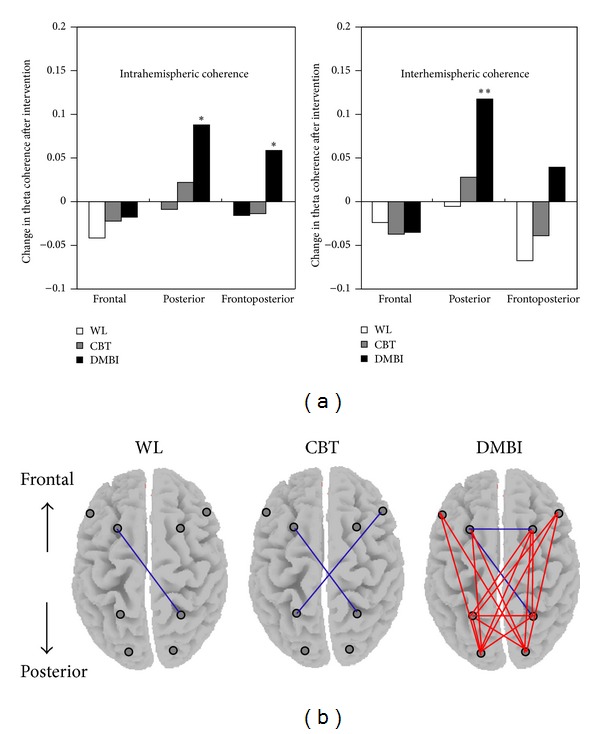
Changes in intra- and interhemispheric theta coherence after intervention across three groups. (a) The bar chart shows the mean difference in theta coherence indices between baseline and postintervention of each group. Positive values indicate increased level of theta coherence after intervention; negative values indicate a reduction in coherence analyzed with paired samples Student's *t*-test; **P < 0.01; *P < 0.05. (b) The topographic map indicates the individual electrode pairs that yield significant changes in theta coherence of each group after intervention. Red lines linking the electrode pairs signify significant coherence increments, whereas blue lines indicate significant coherence decrements analyzed with paired samples Student's *t*-test, *P* ≤ 0.05. WL = waitlist group; CBT = cognitive behavioral therapy group; DMBI = *Dejian* mind-body intervention group.

At baseline, there were no significant differences between the DMBI, CBT, and waitlist groups in the six clusters of intra- and interhemispheric theta coherence; *F* values ranged from 0.025 to 1.522, *P* > 0.05. After intervention, as shown in Figure 3(a), the DMBI group demonstrated significantly enhanced intra- and interhemispheric theta coherence in posterior regions (intraposterior: *t* = 2.116, *P* = 0.025, and effect size = 0.513; interposterior: *t* = 2.629, *P* = 0.009, and effect size = 0.637) and intrahemispheric coherence in frontoposterior brain regions, *t* = 2.039, *P* = 0.029, and effect size = 0.494. In contrast, neither the CBT nor the waitlist group showed similar patterns of elevation of coherence extending through anterior and posterior brain regions: *t* values ranged from −1.664 to 0.675; effect sizes ranged from 0.033 to 0.416, *P* > 0.05. Regarding interhemispheric frontoposterior coherence, the DMBI group also showed a mean increment of 0.039 units, which was significantly greater than that of the CBT group (mean decrement of 0.039 units: *t*(32) = 1.703, *P* = 0.049, and effect size = 0.584) and the waitlist group (mean decrement of 0.068 units: *t*(31) = 2.099, *P* = 0.022, and effect size = 0.728). No significant changes in mean frontal intra- or interhemispheric coherence were observed in any group; *t* values ranged from −0.469 to −1.257; effect sizes ranged from 0.117 to 0.305, *P* > 0.05.

The topographic map in Figure 3(b) illustrates the individual electrode pairs that yielded significant changes in theta coherence after intervention. Consistent with the group mean differences in the clustered coherence indices, the DMBI group exhibited significant coherence elevation at long-range connections between hemispheres that involved anterior-posterior regions and intraposterior regions. Numerous neuroimaging and neuroelectrophysiological findings have suggested that the posterior association cortex and its functional connectivity with the frontal region have a salient role in mediating attention and effortful mental processing [46–49] and that these regions are involved in the pathology of people suffering from attention deficits [50, 51].

### 3.3. Enhanced Frontal Alpha Asymmetry Was Associated with Improvement of Depressive Symptoms

As frontal alpha asymmetry has repeatedly been found to be associated with positive mood, a relaxed state of mind, and depression, we anticipated that the elevated frontal alpha asymmetry that was specifically found in patients who received DMBI might be related to the reduction in their depressive states. Chi-squared tests were performed to explore changes in frontal alpha asymmetry and depression severity (as measured with the BDI-II). Depression severity was as the clinical classifications of the BDI-II. These classifications include “Normal (0–13 points),” “Mild Depression (14–19 points),” “Moderate Depression (20–28 points),” and “Severe Depression (29–63 points).” Participants who were in the normal range at baseline were excluded from analysis unless their condition deteriorated significantly after the intervention. After exclusion, there remained 13, 14, and 14 participants in the waitlist, CBT, and DMBI, respectively. Participants who were classified as having less severe depression after the intervention (e.g., participants who went from “Moderate Depression” at baseline to “Mild Depression” after the intervention) were clustered as “Improved”; all others were clustered as “Not Improved.” Similarly, participants who showed increased alpha asymmetry after the intervention were clustered as “Increased”, whereas those with reduced asymmetry were classified as “Decreased.”

**Figure 4:**
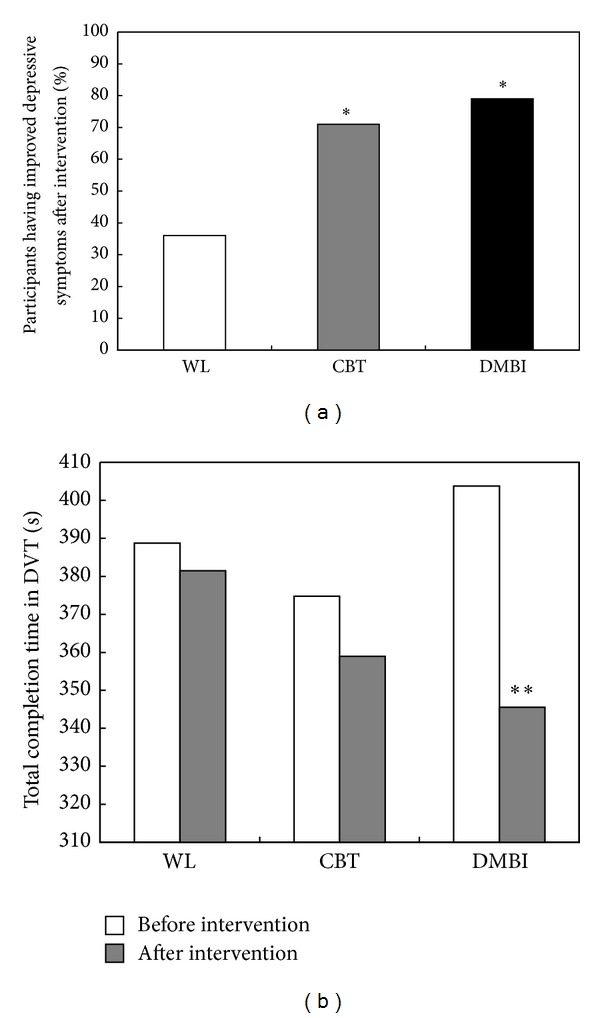
Difference in measures of depressive symptoms and attentional ability at baseline and after 10-week training for waitlist, CBT, and DMBI groups. (a) Each bar represents the percentage of participants in each group having reduced depressive symptoms as indicated by their shift to a less severe clinical classification as measured with the Beck Depression Inventory (BDI-II). (b) The ordinate represents the total completion time of the Digit Vigilance Test (DVT), where shorter completion time indicates greater work efficiency and increased ability to maintain attention on the task. *P < 0.05 (Chi-squared test or paired Student's samples *t*-test).

The results indicated that both DMBI, *χ* ^2^(1) = 5.250, *P* = 0.011, and CBT, *χ* ^2^(1) = 3.590, *P* = 0.029, groups reported significant improvement of depressive symptoms when compared to the waitlist control group (Figure 4(a)). However, the Chi-squared test showed that participants in the DMBI group with improved depressive symptoms were more likely to exhibit increased alpha asymmetry, *χ* ^2^(1) = 6.873, *P* = 0.009 (Table 2). Among the 11 participants with reduced depressive symptoms, 9 (82%) exhibited increased alpha asymmetry after the intervention. Furthermore, all participants who did not exhibit reductions in depression severity exhibited decreases in alpha asymmetry after intervention. Although the CBT group also demonstrated significantly improved postintervention BDI-II scores, no significant association between reduction in depression severity and enhancement of alpha asymmetry was observed, *χ* ^2^(1) = 0.280, *P* = 0.597. As expected, the waitlist group also failed to show any significant association between these two parameters, *χ* ^2^(1) = 1.040, *P* = 0.308.

### 3.4. Enhanced Intra- and Interhemispheric Theta Coherence Was Associated with Attentional Ability

The increased long-range theta coherence in the DMBI group after the 10-session intervention may be suggestive of the possible neural mechanism underlying its improvements in attentional ability, which was revealed by the results of the DVT (Figure 4(b)). There was no significant difference in the total completion time at baseline among the waitlist, CBT, and DMBI groups, *F*(2, 46) = 0.433, *P* = 0.651. After 10-week training, the DMBI group demonstrated significantly improved performance on the DVT attentional task, *t*(15) = 3.623, *P* = 0.002, and effect size = 0.91, when compared to the CBT, *t*(16) = 1.103, *P* = 0.143, and waitlist, *t*(16) = 0.432, *P* = 0.336 groups. Therefore, the substantial improvement on the DVT attention task observed in the DMBI group after the 10-week training suggested that the DMBI had a significant effect on enhancing attentional ability in patients with depression.

To further explore the association between theta coherence and attentional ability, Pearson's correlations were calculated between DVT completion time and mean intra- and interhemispheric frontoposterior/posterior coherence indices after the intervention for each group. DVT completion times were negatively correlated with all four coherence indices in the DMBI group (Table 3); *r* values ranged from −0.41 to −0.57, *P* ≤ 0.05, suggesting that faster response times (which indicate increased attention) were associated with greater levels of coherence. This negative association is consistent with past empirical evidence for the involvement of frontoposterior functional connectivity and the significant contribution of the posterior brain regions in attention processing [46–49]. In contrast, no such associations were found in the CBT or waitlist groups (Table 3).

## 4. Discussion

The present study provides evidence that a Chinese *Chan*-based mind-body intervention can alter brain activity and connectivity patterns that are associated with positive mood and attentiveness in patients with major depressive disorders. It has been widely documented that positive emotions, feelings of well-being, and states of relaxation are associated with greater left-side anterior activation, which is indexed by decreased alpha activity in the left hemisphere [23, 52] and that negative emotion is accompanied by greater right-side activation [44]. While insufficient left-anterior activation and/or excessive right-anterior activation have repeatedly been found in patients with depression [45], enhanced adaptive responses to stressful events have been noted in individuals with greater left-side anterior activation [44]. Our results showed that DMBI was associated with a significant elevation in left-side anterior activation and that the increased left-sided anterior activation was positively correlated with improvements in overall depressive symptoms, as measured by the BDI-II; these findings support our hypothesis that DMBI can elevate positive affect. These results are consistent with the findings of significant alleviations of depressive symptoms and reductions in the intake of antidepressants in patients with depression who practice DMBI [25]. Together, these results indicate that DMBI activates neural networks involved in positive affect and that the therapeutic, depression-reducing effects of DMBI are mediated by enhancement of frontal alpha asymmetry.

Interestingly, although patients who received CBT reported similar improvements in depressive symptoms after the intervention, their increment in frontal alpha asymmetry was not robust enough to reach statistical significance and was about half that of the DMBI group. This finding suggests that CBT has beneficial effects on the subjective feelings of the depressed patients but has relatively minimal effects on neural activity patterns. Although some recent studies have reported CBT-specific enhancements of frontal and/or subcortical region activity, the treatment protocols adopted in those studies typically required 15 to 20 individual sessions or small groups of, at most, 6 participants at a time [53–55]. Therefore, it is plausible that the 10-session CBT protocol and the inclusion of more than 10 persons in the CBT may have precluded results as robust as those reported in previous studies. In contrast, our 10-session DMBI was administered to a group of the same size and was able to foster frontal activation, suggesting that this Chinese *Chan*-based intervention may be a cost-effective complimentary intervention for patients with depression. DMBI's cost-effectiveness is also supported by another previous study that revealed improved depressive mood with increased frontal asymmetry in community-dwelling adults after 4 sessions of DMBI [13].

In addition to the alteration in mood and related neural responses elicited by DMBI, results from this study are also relevant to the electrophysiological changes associated with the attentional network. Our results indicated that DMBI was associated with significant elevations in intra- and interhemispheric frontoposterior and posterior theta coherences. EEG coherence is a correlate of functional connectivity [56, 57] and, in the theta frequency band, coupling between frontal and posterior cortical regions has been shown to underlie attentionally demanding processing [23, 24, 43]. Numerous empirical studies have also suggested the salient role of the posterior cortical regions (particularly the parietal cortex, which is located at the junction of multiple sensory regions and projects to several cortical and subcortical areas) in mediating sustained and selective attention [46, 47]. The robust enhancement in frontoposterior and posterior theta coherence within and between hemispheres in the DMBI group and its association with greater efficiency in a sustained attention task are coherent with the existing scientific findings regarding attention neural networks. The findings of the present study are also consistent with our previous research on the effects of one month of practice of the *Dan Tian *Breathing (a form of *Chan*-based mind-body exercise) in enhancing theta coherence [21]. The increase in theta coherence observed in the present study (maximal increment of 0.12 units) is much greater than that observed in the previous study (maximal increment of 0.04 units). One possible explanation of the more robust results of the present study may be related to the longer duration of intervention in the present study (10 versus 4 sessions). Another possible explanation could be that the present study applied the holistic DMBI model, while the previous study employed only one component of DMBI (i.e., the mind-body exercise). DMBI employs a holistic approach that incorporates integrative treatment of the mind and the body, which, on the one hand, changes the thought process and, on the other hand, improves mental and physical health by refining the diet and encouraging the practice of mind-body exercises for optimal treatment effects.

Collectively, our findings are consistent with the general understanding of the electrophysiological nature of mind-body interventions in which alpha and theta activations have been reported to be associated with changes in affective processes and attentional allocation [23, 58]. Interestingly, we observed both increased anterior left-side activation and increased theta coherence in the DMBI group, indicating that DMBI can produce positive changes in brain function. While the present study has provided some interesting observations on the potential effects of a 10-week DMBI training on improving the mood and cognitive function of patients with depression, yet its long-term effect remains unknown. In addition, it should also be noted that the generalization of the findings to the patient population with depression may be limited by the relatively small sample size. Future studies recruiting a larger sample, with a broader age range, and including longitudinal followup and different clinical populations would help extend current knowledge to a wider population. For example, it will be of interest to reduce stress in patients with anxiety depression or improve attention in children with attention-deficit hyperactivity disorder. In spite of this limitation, DMBI's potential to enhance the functioning of neural structures and the easy-to-practice and cost-effective characteristics of DMBI suggest that this Chinese *Chan*-based mind-body intervention is clinically applicable.

## 5. Conclusion

Our study showed that after 10 sessions of the Chinese* Chan*-based DMBI, patients with depression exhibited significantly increased EEG alpha asymmetry (which is an indication of left-side anterior activation) and intra- and interhemispheric theta coherences in frontoposterior and posterior brain regions. While the alpha asymmetry is an index associated with positive mood, the theta coherence is an index associated with attention. Furthermore, the asymmetry and coherence indices of the DMBI group were correlated with self-reported depression severity levels and performance on an attention test, respectively. In contrast, neither the CBT nor the waitlist group showed significant changes in EEG activity patterns. These findings support the idea that DMBI can promote positive emotional experience and induce a unique state of mind in which relaxation and internalized attention coexist.

## Figures and Tables

**Table 1:** Demographic characteristics of each group of participants.

Characteristics	WL (*n* = 16)	CBT (*n* = 17)	DMBI (*n* = 17)	*F* or *χ* ^2^	*P*
Years of age					
Range	31–62	34–57	32–62	—	—
Mean (SD)	45.44 (8.25)	46.94 (6.54)	47.06 (9.54)	0.198	0.821
Years of education					
Range	3–11	6–16	3–16	—	—
Mean (SD)	8.06 (2.59)	9.82 (2.46)	9.18 (3.13)	1.729	0.189
Gender				1.103	0.576
Male	4	4	2		
Female	12	13	15		

*Note*. WL: waitlist control; CBT: cognitive behavioral therapy; DMBI: *Dejian* mind-body intervention.

**Table 2:** Association between severity level of depression and frontal alpha asymmetry.

Subgroups of change in alpha asymmetry^a^	Subgroups of change in severity level of depression^b^		*χ* ^2^	E.S.
	Improved	Not improved		
WL (*n* = 13)			1.040	0.28
Increased	1 (8%)	5 (39%)		
Decreased	3 (23%)	4 (31%)		
CBT (*n* = 14)			0.280	0.14
Increased	6 (43%)	3 (21%)		
Decreased	4 (29%)	1 (7%)		
DMBI (*n* = 14)			6.873**	0.70^++^
Increased	9 (64%)	0 (0%)		
Decreased	2 (14%)	3 (21%)		

*Note*. WL: waitlist control; CBT: cognitive behavioral therapy; DMBI: *Dejian* mind-body intervention; E.S.: effect size as measured by Phi value.

^a^Subgroups of change in alpha asymmetry are determined by the change in frontal alpha asymmetry from baseline to postintervention, where higher asymmetry level after intervention is considered as “Increased,” and vice versa.

^b^Subgroups of change in severity level of depression are determined by the change in clinical classification based on the total score of Beck Depression Inventory from baseline to postintervention, where movement from more severe to less severe category after intervention is considered as “Improved”; otherwise, it is “Not Improved.”

**P < 0.01; ^++^large effect size.

**Table 3:** Correlation between digit vigilance test performance and theta coherence indices.

Coherence indices	Completion time of Digit Vigilance Test		
	WL (*n* = 16)	CBT (*n* = 17)	DMBI (*n* = 17)
Intrahemisphere			
Frontoposterior	−0.232	0.175	−0.568**
Posterior	−0.030	0.439*	−0.437*
Interhemisphere			
Frontoposterior	−0.231	0.133	−0.536*
Posterior	0.031	0.334	−0.412*

*Note*. WL: waitlist control; CBT: cognitive behavioral therapy; DMBI: *Dejian* mind-body intervention.

*P ≤ 0.05; **P < 0.01.

## References

[1] Deckro GR, Ballinger KM, Hoyt M (2002). The evaluation of a mind/body intervention to reduce psychological distress and perceived stress in college students. *Journal of American College Health*.

[2] Stetter F, Kupper S (2002). Autogenic training: a meta-analysis of clinical outcome studies. *Applied Psychophysiology Biofeedback*.

[3] Shapiro D, Cook IA, Davydov DM, Ottaviani C, Leuchter AF, Abrams M (2007). Yoga as a complementary treatment of depression: effects of traits and moods on treatment outcome. *Evidence-Based Complementary and Alternative Medicine*.

[4] Naliboff BD, Fresé MP, Rapgay L (2008). Mind/body psychological treatments for irritable bowel syndrome. *Evidence-Based Complementary and Alternative Medicine*.

[5] Caudill M, Schnable R, Zuttermeister P, Benson H, Friedman R (1991). Decreased clinic utilization by chronic pain patients after behavioral medicine intervention. *Pain*.

[6] Yeh GY, Wood MJ, Lorell BH (2004). Effects of *Tai Chi* mind-body movement therapy on functional status and exercise capacity in patients with chronic heart failure: a randomized controlled trial. *American Journal of Medicine*.

[7] Tsang HWH, Fung KMT, Chan ASM, Lee G, Chan F (2006). Effect of a qigong exercise programme on elderly with depression. *International Journal of Geriatric Psychiatry*.

[8] Tsang HWH, Mok CK, Au Yeung YT, Chan SYC (2003). The effect of Qigong on general and psychosocial health of elderly with chronic physical illnesses: a randomized clinical trial. *International Journal of Geriatric Psychiatry*.

[9] Sandlund ES, Norlander T (2000). The effects of *tai chi chuan* relaxation and exercise on stress responses and well-being: an overview of research. *International Journal of Stress Management*.

[10] Wang C, Collet JP, Lau J (2004). The effect of *tai chi* on health outcomes in patients with chronic conditions. *Archives of Internal Medicine*.

[11] Chan AS (2010). *The Shaolin Chanwuyi: A Chinese Chan Buddhism*.

[12] Chan AS (2013). *Contemporary Application of Shaolin Medicine: Dejian Mind-Body Intervention*.

[13] Chan AS, Cheung M-C, Tsui WJ, Sze SL, Shi D (2011). Dejian mind-body intervention on depressive mood of community-dwelling adults: a randomized controlled trial. *Evidence-Based Complementary and Alternative Medicine*.

[14] Chan AS, Sze SL, Shi D (2008). Traditional Chinese mind-body exercises improve self control ability of an adolescent with Asperger’s disorder. *Journal of Psychology in Chinese Societies*.

[15] Chan AS, Sze SL, Cheung M-C, Lam JMK, Shi D (2009). Dejian mind-body intervention improves the functioning of a patient with chronic epilepsy: a case report. *Cases Journal*.

[16] Chan AS, Sze SL, Cheung M-C, Han YMY, Leung WWM, Shi D (2011). *Dejian* mind-body intervention improves the cognitive functions of a child with autism. *Evidence-Based Complementary and Alternative Medicine*.

[17] Chan AS, Sze SL, Han YMY, Cheung MC (2012). A *Chan* dietary intervention enhances executive functions and anterior cingulate activity in autism spectrum disorders: a randomized controlled trial. *Evidence-Based Complementary and Alternative Medicine*.

[18] Chan AS, Han YMY, Cheung MC, Patel VB, Preedy VR, Martin CR (2013). Chinese *Chan*-based prospective neuropsychological intervention for autistic children. *The Comprehensive Guide to Autism*.

[19] Chan AS, Sze SL, Siu NY, Lau EM, Cheung MC (2013). A Chinese mind-body exercise improves self-control of children with autism: a randomized controlled trial. *PLoS ONE*.

[20] Jacobs GD, Friedman R (2004). EEG spectral analysis of relaxation techniques. *Applied Psychophysiology Biofeedback*.

[21] Chan AS, Cheung M-C, Sze SL, Leung WW-M, Shi D (2011). *Shaolin Dan Tian* breathing fosters relaxed and attentive mind: a randomized controlled neuro-electrophysiological study. *Evidence-Based Complementary and Alternative Medicine*.

[22] Davidson RJ, Kabat-Zinn J, Schumacher J (2003). Alterations in brain and immune function produced by mindfulness meditation. *Psychosomatic Medicine*.

[23] Aftanas LI, Golocheikine SA (2001). Human anterior and frontal midline theta and lower alpha reflect emotionally positive state and internalized attention: high-resolution EEG investigation of meditation. *Neuroscience Letters*.

[24] Sauseng P, Hoppe J, Klimesch W, Gerloff C, Hummel FC (2007). Dissociation of sustained attention from central executive functions: local activity and interregional connectivity in the theta range. *European Journal of Neuroscience*.

[25] Chan AS, Wong QY, Sze LS, Kwong PPK, Han YMY, Cheung MC (2012). A Chinese *Chan*-based mind-body intervention for patients with depression. *Journal of Affective Disorders*.

[26] Chan AS, Wong QY, Sze LS, Kwong PPK, Han YMY, Cheung MC (2012). A Chinese *Chan*-based mind-body intervention improves sleep on patients with depression: a randomized controlled trial. *The Scientific World Journal*.

[27] Butler AC, Chapman JE, Forman EM, Beck AT (2006). The empirical status of cognitive-behavioral therapy: a review of meta-analyses. *Clinical Psychology Review*.

[28] Shelton RC (2009). Long-term management of depression: tips for adjusting the treatment plan as the patient’s needs change. *The Journal of Clinical Psychiatry*.

[29] American Psychiatric Association (2000). *Diagnostic and Statistical Manual of Mental Disorders*.

[30] So E, Kam I, Leung CM, Chung D, Liu Z, Fong S (2003). The Chinese-bilingual SCID-I/P project: stage 1—reliability for mood disorders and schizophrenia. *Hong Kong Journal of Psychiatry*.

[31] Cuijpers P, van Straten A, Andersson G, van Oppen P (2008). Psychotherapy for depression in adults: a meta-analysis of comparative outcome studies. *Journal of Consulting and Clinical Psychology*.

[32] Beck AT, Steer RA, Brown GK (1996). *Manual For the Beck Depression Inventory-II*.

[33] Chang H (2007). Depressive symptom manifestation and help-seeking among Chinese college students in Taiwan. *International Journal of Psychology*.

[34] Lewis RF (1995). *Digit Vigilance Test: Professional User’s Guide*.

[35] Jasper HH (1958). The 10–20 electrode system of the international federation. *Electroencephalography and Clinical Neurophysiology*.

[36] Beck AT, Rush AJ, Shaw BF, Emery G (1987). *Cognitive Therapy of Depression*.

[37] Greenberger D, Padesky C (1995). *Mind over Mood: Change How You Feel by Changing the Way You Think*.

[38] Bieling PJ, McCabe RE, Antony MM (2006). *Cognitive-Behavioural Therapy in Groups*.

[39] Jacobson E (1938). *Progressive Relaxation*.

[40] Gaylord C, Orme-Johnson D, Travis F (1989). The effects of the transcendental mediation technique and progressive muscle relaxation on EEG coherence, stress reactivity, and mental health in black adults. *International Journal of Neuroscience*.

[41] Klimesch W (1999). EEG alpha and theta oscillations reflect cognitive and memory performance: a review and analysis. *Brain Research Reviews*.

[42] Pivik RT, Broughton RJ, Coppola R, Davidson RJ, Fox N, Nuwer MR (1993). Guidelines for the recording and quantitative analysis of electroencephalographic activity in research contexts. *Psychophysiology*.

[43] Womelsdorf T, Fries P (2007). The role of neuronal synchronization in selective attention. *Current Opinion in Neurobiology*.

[44] Davidson RJ (2000). Affective style, psychopathology, and resilience: brain mechanisms and plasticity. *American Psychologist*.

[45] Henriques JB, Davidson RJ (1990). Regional brain electrical asymmetries discriminate between previously depressed and healthy control subjects. *Journal of Abnormal Psychology*.

[46] Behrmann M, Geng JJ, Shomstein S (2004). Parietal cortex and attention. *Current Opinion in Neurobiology*.

[47] Han S, Jiang Y, Gu H (2004). The role of human parietal cortex in attention networks. *Brain*.

[48] Sarnthein J, Petsche H, Rappelsberger P, Shaw GL, von Stein A (1998). Synchronization between prefrontal and posterior association cortex during human working memory. *Proceedings of the National Academy of Sciences of the United States of America*.

[49] Sarter M, Givens B, Bruno JP (2001). The cognitive neuroscience of sustained attention: where top-down meets bottom-up. *Brain Research Reviews*.

[50] Ojeda N, Ortuño F, Arbizu J (2002). Functional neuroanatomy of sustained attention in schizophrenia: contribution of parietal cortices. *Human Brain Mapping*.

[51] Silk T, Vance A, Rinehart N (2005). Fronto-parietal activation in attention-deficit hyperactivity disorder, combined type: functional magnetic resonance imaging study. *The British Journal of Psychiatry*.

[52] Urry HL, Nitschke JB, Dolski I (2004). Making a life worth living: neural correlates of well-being. *Psychological Science*.

[53] Fu CHY, Williams SCR, Cleare AJ (2008). Neural responses to sad facial expressions in major depression following cognitive behavioral therapy. *Biological Psychiatry*.

[54] Ritchey M, Dolcos F, Eddington KM, Strauman TJ, Cabeza R (2011). Neural correlates of emotional processing in depression: changes with cognitive behavioral therapy and predictors of treatment response. *Journal of Psychiatric Research*.

[55] Yoshimura S, Okamoto Y, Onoda K (2013). Cognitive behavioural therapy for depression changes medial prefrontal and ventral anterior cingulate cortex activity associated with self-referential processing. *Social Cognitive and Affective Neuroscience*.

[56] Murias M, Webb SJ, Greenson J, Dawson G (2007). Resting state cortical connectivity reflected in EEG coherence in individuals with autism. *Biological Psychiatry*.

[57] Rippon G, Brock J, Brown C, Boucher J (2007). Disordered connectivity in the autistic brain: challenges for the ’new psychophysiology’. *International Journal of Psychophysiology*.

[58] Cahn BR, Polich J (2006). Meditation states and traits: EEG, ERP, and neuroimaging studies. *Psychological Bulletin*.

